# Results of coronary artery spasm treatment after cardiac surgery procedures

**DOI:** 10.1186/1749-8090-10-S1-A51

**Published:** 2015-12-16

**Authors:** DS Tungusov, II Chernov, DG Tarasov, AV Pavlov, DA Kondratyev, RA Urtaev, VV Pasyuga

**Affiliations:** 1 Federal Centre for Cardiovascular Surgery, Astrakhan, 414024, Russia

## Background/Introduction

Postoperative coronary artery spasm (CAS) is an infrequent life-threatening event after cardiac surgery. We have suggested to cross clamp aorta for short time and to inject nitrates into the aortic root in case of suspicion of CAS. It enable us to deliver nitrates especially into coronary arteries during operation (patent RU 2552890). Another method is conventional coronary angiography with injection of nitrates.

## Aims/Objectives

The purpose of this study was to compare two methods of treatment CAS.

## Method

Between January 2013 and December 2014 we prospectively collected data from a consecutive cohort of 3452 patients who underwent cardiac surgery. CAS was registered in 60 (1,7%) patients and was associated with new ST-segment changes on electrocardiogram, ventricular arrhythmias, cardiac arrest or hemodynamic collapse, new changes in regional wall motion, or any other relevant suspect of myocardial ischemia during operation and postoperative intensive care unit stay were included. Patients with non-patent grafts and with abnormal coronary blood flow surgically caused were excluded. The method of CAS treatment was based on surgeon decision.

## Results

22 patients underwent coronary angiography after operation. Injection of nitrates was performed into coronary arteries or grafts (group 1). In 38 cases injection of nitrates into aortic root was made on short time cross clamp (group 2). Perioperative myocardial infarction (MI) was found in 13 (59.9%) cases (group 1), 6 (15.8%) cases (group 2) (p < 0.0001). A significant difference was observed between the two groups in the length of hospitalization (p < 0.05), length of ICU stay (p < 0.05). The in-hospital mortality in the first group was 2 (9.1%) in the second group the absence of mortality was registered.

## Discussion/Conclusion

Early suspicion of CAS and intraoperative injection of nitrates into aortic root enables to decrease MI, in-hospital mortality, ICU and in-hospital stay.

